# Establishment of long-term ostracod epidermal culture

**DOI:** 10.1007/s11626-020-00508-8

**Published:** 2020-10-09

**Authors:** Siân R. Morgan, Laura Paletto, Benjamin Rumney, Farhana T. Malik, Nick White, Philip N. Lewis, Andrew R. Parker, Simon Holden, Keith M. Meek, Julie Albon

**Affiliations:** 1 School of Optometry and Vision Sciences, Cardiff University, Cardiff, CF24 4HQ UK; 2 Cardiff Institute for Tissue Engineering and Repair, School of Pharmacy and Pharmaceutical Sciences, Cardiff University, Redwood Building, King Edward VII Avenue, Cardiff, CF10 3NB UK; 3 Vivat Scientia Bioimaging Laboratories, School of Optometry and Vision Sciences, Cardiff University, Cardiff, CF24 4HQ UK; 4 Lifescaped, Somerset House, London, WC2R 1LA UK; 5 Green Templeton College, University of Oxford, Woodstock Road, Oxford, OX2 6HD UK; 6 Defence Science and Technology Laboratory (DSTL), Porton Down, Salisbury, SP4 0JQ UK

**Keywords:** Crustacean, Ostracod, Carapace, Epidermal, Culture

## Abstract

Primary crustacean cell culture was introduced in the 1960s, but to date limited cell lines have been established. *Skogsbergia lerneri* is a myodocopid ostracod, which has a body enclosed within a thin, durable, transparent bivalved carapace, through which the eye can see. The epidermal layer lines the inner surface of the carapace and is responsible for carapace synthesis. The purpose of the present study was to develop an in vitro epidermal tissue and cell culture method for *S. lerneri*. First, an optimal environment for the viability of this epidermal tissue was ascertained, while maintaining its cell proliferative capacity. Next, a microdissection technique to remove the epidermal layer for explant culture was established and finally, a cell dissociation method for epidermal cell culture was determined. Maintenance of sterility, cell viability and proliferation were key throughout these processes. This novel approach for viable *S. lerneri* epidermal tissue and cell culture augments our understanding of crustacean cell biology and the complex biosynthesis of the ostracod carapace. In addition, these techniques have great potential in the fields of biomaterial manufacture, the military and fisheries, for example, in vitro toxicity testing.

## Introduction

Myodocopid ostracods (or ‘seed shrimps’) are a suborder of small marine crustaceans of length between 1 and 32 mm, which pass through a series of six, juvenile growth stages known as instars before reaching the adult stage (McKenzie *et al*. 1999). Their shrimp-like bodies are suspended within a relatively thin (20–60 μm), bivalved calcified carapace by ‘central adductor muscles’, which run through the body attached to each valve; a hinge joins the two valves enabling them to open and close their carapace while preserving its rigidity (McKenzie *et al*. 1999). The majority of myodocopid ostracods are benthic; they bury up to 1 cm depth in the sediments of the ocean floor (McKenzie *et al*. 1999). Most myodocopid ostracods can be distinguished from other ostracods as they possess good vision with well-developed compound eyes (Parker *et al*. 2019), up to one third of their overall body size (Parker 1998). Some myodocopids in the family Cypridinidae are known to possess transparent carapaces or transparent windows in the parts of the carapace that cover the eyes (Parker *et al*. 2019). The cypridinid species used in the present study, *Skogsbergia lerneri*, is 2–3 mm in length and found at a depth of 1–130 m along the eastern coast of the American continents from Florida to the top of Brazil and the Caribbean islands (Kornicker 1958) and possess a carapace with uniform transparency.

The carapace structure comprises four layers: a thin outer layer devoid of chitin known as the epicuticle; a dense laminar layering of chitin forming the exocuticle; a less dense laminar layer known as the endocuticle and the epidermal cell layer (Stevenson 1985). This epidermal layer is essential to the formation of the carapace; it shows intense secretory activity and orchestrates secretion of the other layers (Stevenson 1985; Mrak *et al*. 2015). In addition, the epidermal cells are essential for the reabsorption of calcium from preceding layers just before ecdysis (or moulting) to store minerals (Glötzner and Ziegler 2000). Ecdysis involves the shedding and formation of old to new exoskeleton and is a fundamental process that is essential for growth of all crustaceans. Understanding the complexity of creation and assembly of the thin, highly transparent, yet presumably strong and durable ostracod carapace would provide huge potential for material manufacture, with numerous applications in the biomaterials industry, as well as facilitating marine *in vitro* toxicity testing.

To date, crustacean cell culture has proved problematic and is limited (Toullec 1999; Jayesh *et al*. 2012). The purpose of the present study was to develop an epidermal tissue and cell culture method for *S. lerneri*. To achieve this aim, first an optimal environment for the growth and viability of this cell type was determined, while maintaining sterility and proliferative potential. Next, a microdissection technique to remove the epidermal layer for explant culture was established and finally, a cell dissociation process for cell culture was determined.

## Materials and Methods

### Source of Tissue

*Skogsbergia lerneri* ostracods used in the present study were collected biannually off the coast of Florida following the acquisition of Special Activity licenses (SAL-16-1796-SR and SAL-19-1796-SR) and Florida Keys National Marine Sanctuary permits (FKNMS-2016-116 and FKNMS-2018-116). The ostracods were then transported to Cardiff University, where, after conditioning to aquaculture conditions, they were introduced into aquaculture within purpose-built aquarium tanks containing artificial seawater made up to a salinity of 35% using Pro-Reef Sea Salt (Tropic Marin, Wartenberg, Germany) with a pH 7.5, and maintained at 25–26°C to simulate the seawater conditions of their local habitat in the Florida Keys (water had been previously tested by the collectors). Twice weekly, the ostracods were fed with Whitebait fish obtained from a local tackle centre (Garry Evan Ltd, Cardiff, UK). The quantities of tank water were exchanged with fresh artificial seawater, biweekly, or more if required based on the testing of levels of nitrites, nitrates and ammonium using test kits (Tropic Marin, Wartenberg, Germany) to ensure an optimal environment for the ostracods. Ostracods were maintained in these aquaculture conditions for the duration of their lifecycle or until required for experimentation.

### Ostracod Dissection and Removal of Carapace

**Figure 1.**
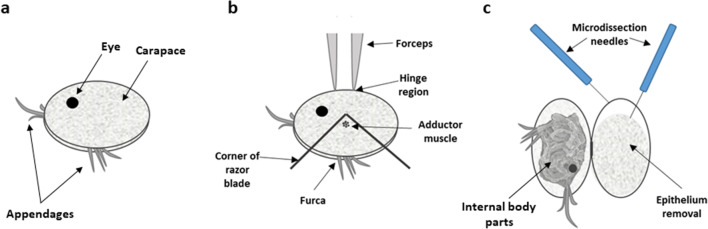
Ostracod dissection and carapace epidermal removal. (*a*) A whole ostracod indicating the compound eye, closed carapace and appendages (antennule, antenna and furca). (*b*) The ostracod is stabilised at the hinge and a single-edged razor blade is inserted into the opening at the furca to cut through the adductor muscle. (*c*) The carapace is opened and the epithelium is removed using fine microdissection needles.

*Skogsbergia lerneri* were removed from the aquarium, anaesthetised in ice-cold sterile 0.9% saline for 2 min and euthanised by immersion in 30% ethanol for 2 min which relaxed the adductor muscle. To isolate the carapace, ostracod valves were opened, under a dissection microscope, by inserting the corner of a single-edged razor blade into the opening at the caudal furca, and the adductor muscle was severed (Fig. 1). While stabilised by forceps at the hinge region, the carapace was opened and internal body parts were removed. The carapace, with or without epidermal layer, was carefully dissected away using microdissection needles.

### X-Ray Microtomography

In order to understand ostracod carapace microstructure, prior to microdissection described above, X-ray microtomography was performed on adult ostracods on Beamline I13 (MT17839-1), at the Diamond Light Source national synchrotron facility (Didcot, UK). This enabled rapid, non-destructive, three-dimensional high spatial and contrast resolution imaging of the *S. lerneri* structure and components. Whole adult *S. lerneri* ostracods (*n* = 3) or single carapace valves (*n* = 3) were mounted onto the metal pin of sample holders (CN Technical Services Ltd, Wisbech, UK) using super glue (Loctite, Westlake, OH).

Raw images were collected using a monochromatic beam and pco.edge 5.5 detector. Samples were scanned through 180°, using a 0.5-s acquisition time at ×4 total magnification and a 12 cm sample-to-detector distance for whole ostracods. For carapace valves, scans were performed at a ×10 total magnification using a 0.2-s acquisition time and a 22.5-cm sample-to-detector distance. Raw data were reconstructed using a series of tomocommands and ImageJ, and then rendered using Avizo 9 software.

### Optimisation of Ostracod Microdissection for Epidermal Culture

Ostracod microdissection was performed, as described above, in ice-cold sterile saline under a dissection microscope. Next, carapace halves were decontaminated prior to placement in culture. Following dissection, carapace halves were incubated in 2.5 μg/ml amphotericin B (Sigma, Gillingham, UK) and/or 2% povidone–iodine in saline (Sigma) for 0–4 min (*n* = 10 per disinfectant tested at each time point). Cell viability, expressed as a percentage, was determined by quantitation of live (green fluorescence) and dead cells (red fluorescence) using a live/dead assay kit (Abcam, Cambridge, UK, ab115347) in images captured using a Leica 6000 fluorescent microscope and Leica Application Suite X (LAS X) software.

For carapace-epithelial culture, the epithelial layer was retained on the carapace. For epidermal explant culture, the epidermal layer was carefully dissected away using microdissection needles (Fig. 1).

### Optimisation of Medium for Epidermal Culture

To determine the optimal combination of culture medium and supplements for epidermal cell culture, carapace-epithelia (i.e. valve with epidermal layer intact, *n* = 15 per culture condition) and/or epidermal explants (*n* = 15 per culture condition) of later-stage lifecycle *S. lerneri* (instar 4–adult) were cultured in various media/supplement combinations at ambient temperature (20°C), 25 or 37°C, either in air or 5% CO_2_. These temperatures were chosen as a starting point to establish ostracod epidermal cultures based on successful cell and/or tissue culture described for marine fish (ambient room temperature; Nicholson 1989), invertebrates (25–30°C; Hink 1979) and also the seawater temperature of their local habitat at the Florida Keys. In addition, among the culture media tested (see below) were those often used for mammalian cell culture at 37°C. Although this temperature is high for crustacean cells, it has been used to establish fish cell cultures (Shima *et al*. 1980; Nicholson 1989), so it was included.

Culture media included Leibovitz-15 (L-15) (Sigma), medium 199 (M199) (Sigma), DMEM (high and low glucose) (Thermo Fisher Scientific, Loughborough, UK), Grace’s (Sigma), Schneider’s (Sigma) and Hink’s TNM-FH (Sigma) insect media. Supplements, previously proposed to enhance crustacean cultures (Chen *et al*. 1986; Luedeman and Lightner 1992; Hsu *et al*. 1995; Frerichs 1996; Kasornchandra *et al*. 1999; Toullec 1999; Walton and Smith 1999; Mulford *et al*. 2001; Shimizu *et al*. 2001; Lang *et al*. 2002a; Maeda *et al*. 2003), in different combinations and/or concentrations, included heat-inactivated foetal bovine serum (FBS, 5, 10 or 20%; Sigma), amphotericin B (2.5 or 0.25 μg/ml), penicillin–streptomycin (100 or 200 μg/ml; Sigma), GlutaMAX (4 or 2 mM; Sigma), MEM non-essential amino acids (1/1000; Gibco, Fisher Scientific, Loughborough, UK), 10 mg/ml copper II sulphate (Sigma), 10 ng/ml epidermal growth factor (EGF; Sigma), 20 ng/ml fibroblast growth factor-basic (FGF; Sigma), 10% salt solution (sodium citrate, Sigma; calcium chloride, Sigma; magnesium chloride, Sigma), 10 mM HEPES (Sigma), 2 mM l-glutamine (Sigma) and sodium chloride (5 or 6 g/l; Sigma).

In addition, to promote epidermal adhesion, epidermal explants were cultured on different substrates (plastic, fibronectin (Sigma), collagen type I (Corning, Corning, New York)) on 12 or 24 multi-well plates (Starlab, Milton Keynes, UK), culture inserts (Corning) and/or 35-mm culture dishes (Corning). Percentage cell viability of epidermal explants cultures (up to 4 wk) and carapace-epithelia cultures (up to 3 wk) was evaluated as described above, with Hoechst 33342 nuclear counterstaining (Molecular Probes, Life Technologies, Loughborough, UK).

### Epidermal Cell Proliferation in Carapace-Epithelia–EdU Incorporation

Cell proliferation in ostracod epidermal cells (*n* = 5 carapace-epithelial cultures) was assayed using a Baseclick EdU HTS Kit (Sigma). EdU (5-ethynyl-2′-deoxyuridine) was incorporated into the DNA of cycling cells at the beginning of culture by addition of medium containing EdU for 24 and 72 h. The epidermal cells were fixed in 4% paraformaldehyde for 15 min, then permeabilised using 0.1% Triton X-100 in PBS for 10 min, before incubation in the click assay mixture (reaction buffer, catalyst solution, 5-TAMRA-PEG3-Azide dye (excitation = 546 nm, emission = 579 nm) and buffer additive) for 30 min in the dark, at room temperature. Cell nuclei were counterstained with Hoechst 33342 (Molecular Probes, Life Technologies) and visualised using fluorescence microscopy for calculation of percentage proliferating cells.

### Epidermal Cell Proliferation in Carapace-Epithelia–BrdU Incorporation

BrdU immunolocalisation was assayed in cultured intact carapace-epithelia (*n* = 5) following BrdU incorporation *in vitro* over 48 h in a 10 μM BrdU (ab142567; Abcam) labelling solution. The samples were prepared, fixed and permeabilised as described above for EdU incorporation, then incubated in 1 M HCl for 30 min at room temperature. The samples were then blocked in 5% normal goat serum (ab156046; Abcam) in PBS for 1 h, incubated in rabbit polyclonal anti-BrdU (1/100 dilution, Abcam, ab152095) overnight at 4°C and then rinsed three times in 1× PBS. After an incubation for 2 h in the dark at room temperature in goat anti-rabbit IgG H&L conjugated to Alexa Fluor 555 (1/1000 dilution, ab150086, Abcam), the samples were mounted in ProLong Gold Anti-fade mountant with DAPI (Thermo Fisher Scientific) and visualised using fluorescent microscopy. Percentage cell proliferation was quantified.

### Transmission Electron Microscopy of Cultured Ostracod Epidermal Tissue

Cultured epidermal tissue on adult ostracod carapaces at three culture time points (0, 1 and 2 wk, *n* = 3 at each time point) were removed from culture and incubated at 4°C overnight in Karnovsky fixative (2% paraformaldehyde, 2.5% glutaraldehyde, in 0.1 M cacodylate buffer). Fixed carapace-epidermal valves were transferred to 1.5% potassium ferricyanide/1% osmium tetroxide in 0.1 M cacodylate buffer for 1 h and washed in distilled water, before 1-h immersions in 1% aqueous osmium tetroxide and 1% aqueous uranyl acetate, with thorough washing in distilled water after each. After an additional 1-h incubation in Walton’s lead aspartate (Walton 1979) at 60°C with further washes, samples were dehydrated through serial ethanols to 100%, immersed in propylene oxide, before Durcapan resin infiltration and embedding over 2 d. The resin was hardened at 60°C for 48 h and ultrathin sections (∼100 nm thick) were cut and viewed on G300 copper grids in a JEM 1010 transmission electron microscope (Jeol, Welwyn Garden City, UK).

### Optimisation of Epithelial Cell Dissociation

To determine the most effective enzyme for dissociation of the epidermal cells, a number of enzymes were tested (see Table 1) on dissected epidermal layers from later-stage lifecycle *S. lerneri* ostracods (instar 4–adult, *n* = 30 ostracods per enzyme protocol). Of all enzymes tested, Dispase I (Sigma) at a concentration from 0.6 to 1.0 U/ml, dissolved in M199 (DPBS) at 25°C was found to be optimal, based on successful cell dissociation of epidermal tissue into discrete cells in a 30-min incubation, while maintaining cell viability.

**Table 1:** Enzymatic dissociation of ostracod epidermal cells. A number of protocols were tested, with optimal cell dissociation achieved following incubation in 0.6–1.0 U/ml Dispase I in M199 at 25°C for 30 min

Enzyme/enzyme combination	Temperature (°C)	Duration
0.04% EDTA in DPBS	25	30 min, 2 h, 2.5 h
0.04% EDTA + Dispase II (2.4 U/ml)	25	1 h, 2 h, 3 h
Accutase	Room temperature, 25, 37	30 min, 10 min, 20 min, 1 h
Accumax	25	30 min, 1 h
0.25% Trypsin/EDTA	25	20 min, 1 h
0.05% Trypsin/EDTA	25, 37	20 min, 1 h, 2 h
0.15% Collagenase I	25	2 h, 2.5 h
Collagenase I (200 U/ml) + Dispase II (2.4 U/ml) + 3 mM CaCl_2_ in DPBS	25, 30, 37	30 min, 2 h
Dispase II in DPBS (2.4 U/ml)	25	1 h, 2 h
Dispase I in DPBS (0.6–1.0 U/ml)	25 25	30 min 1 h
Dispase I in M199 (0.6–1.0 U/ml)	25 25	30 min 1 h
0.05% Trypsin/EDTA + Dispase II (2.4 U/ml)	25 37	1 h, 2 h, 2.5 h 1 h, 2 h, 2.5 h
0.25% Trypsin/EDTA + collagenase I (200 U/ml)	25 37	1 h, 2 h 1 h, 2 h
0.05% Trypsin/EDTA + collagenase I (200 U/ml)	25 37	1 h, 2 h 1 h, 2 h
0.05% Trypsin/EDTA + collagenase I (200 U/ml) + 3 mM CaCl_2_	37	4 h
Accutase ➔ 0.05% Trypsin/EDTA + collagenase I (200 U/ml) + 3 mM CaCl_2_	Room temperature ➔ 37	20 min ➔ 2 h
0.25% Collagenase IV	25	30 min
Dispase II (2.4 U/ml) + 0.25% collagenase IV	37	1 h
Dispase II (2.4 U/ml) + 0.04% EDTA + 0.25% collagenase IV + collagenase I (200 U/ml)	37	1 h

To further optimise the cell dissociation protocol using Dispase I, epidermal tissue was pooled from 30 ostracods for each protocol tested (triplicate samples for each) which included incubations in Dispase I in M199 (0.6–1.0 U/ml) at 25°C for 5, 10, 20, 30 or 40 min.

The dissociated cells and medium were then passed through an 8-μm filter (MF-Millipore, Sigma) to separate the dissociated cells from any residual tissue remnants. Next, the dissociated cells were thoroughly, but gently, washed in M199 medium, within wells containing 4-μm inserts to fully remove enzyme, while avoiding loss of cells. The cells were then cultured on inserts on coated wells in M199 containing 2 mM GlutaMAX, 10% non-essential amino acids, 200 μg/ml penicillin streptomycin and 0.25 μg/ml amphotericin B for 0, 1, 2 and 3 d. Cell viability, quantified as a percentage, was assessed at each time point, by using a live/dead assay kit (Abcam, ab115347), as described above for epidermal culture.

### Determination of Substrate for Epidermal Cell Culture

Using optimal conditions for dissociation of the epidermal cells (incubation in Dispase I in M199 (0.6–1.0 U/ml) for 10 min), ostracod epidermal cells were plated on different substrates (i.e. cells were pooled from 30 ostracods per substrate-coated well) to promote adherence during culture in the optimised M199 medium described above. Substrates included collagen type I (48-well; Sigma), collagen type IV (48-well; Sigma), laminin (24-well; Corning), fibronectin (24-well, BioCoat; Corning), Matrigel (48-well; Sigma) and chitosan. For chitosan coatings, 4% (*w*/*v*) chitosan (>90% degree deacetylation; Sigma) in 2% (*v*/*v*) acetic acid was autoclaved, applied to 48-well plates and allowed to dry for 24 h to form a thin film. The acidity of the films was neutralised with 0.5 M NaOH and washed repeatedly with Hank’s balanced salt solution until film pH returned to a physiological range (pH = 7.4).

### Proliferation of Dissociated Epidermal Cells in Culture

Epidermal cell proliferation of cultured ostracod epidermal cells was assessed after 7 d of culture in optimised M199 medium, described above, on substrates collagen type I (*n* = 3 cultures) or IV (*n* = 3 cultures). The latter were deemed optimal substrates for epidermal cell adherence, viability and spreading (see ‘Results’ section). In brief, following 7 d in culture, cells were incubated in 10 μM BrdU culture medium for 24 h and assayed as described above.

## Results

### X-Ray Microtomography Reconstructions

**Figure 2.**
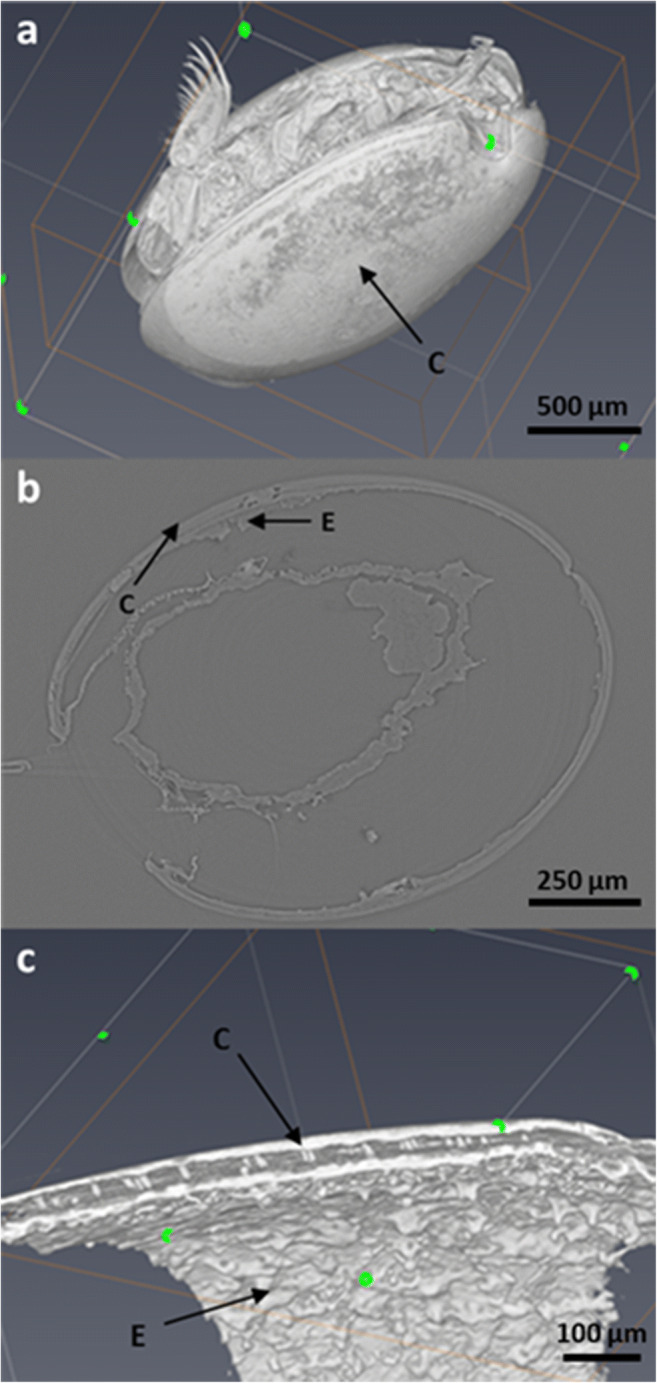
X-Ray microtomography reconstruction images of an adult ostracod used to understand ostracod carapace microstructure, prior to microdissection. (*a*) A whole adult ostracod rotated to reveal the inner body parts and appendages through the furca region of the partially open carapace. (*b*) Microtomography image slice at a depth of 810 μm from within a 3D image stack of an ostracod. (*c*) A cross-section of the ostracod bivalve displaying the layering of the carapace. *C* denotes ostracod carapace and *E* denotes underlying epidermal cell layer.

X-Ray microtomography 3D reconstructions of whole adult *S. lerneri* ostracods (*n* = 3) and single carapace valves (*n* = 3) were rendered to produce high spatial and contrast resolution images that were used to provide information on ostracod microstructure, prior to microdissection (Fig. 2). The 3D images revealed the furca region opening of the carapace, through which the corner of a razor blade could be inserted and the adductor muscle severed, facilitating effective dissection. Image slices (example shown in Fig. 2*b*) from within a 3D image stack were used to make thickness measurements of the ostracod carapace and epidermal cell layer, in order to get an idea of scale. The carapace and epidermal cell layer thickness in these ostracods ranged from 13.67 to 17.09 μm and 12.48–13.88 μm, respectively.

### Epidermal Tissue Culture–Microdissection and Disinfection Protocols Prior to Culture

**Figure 3.**
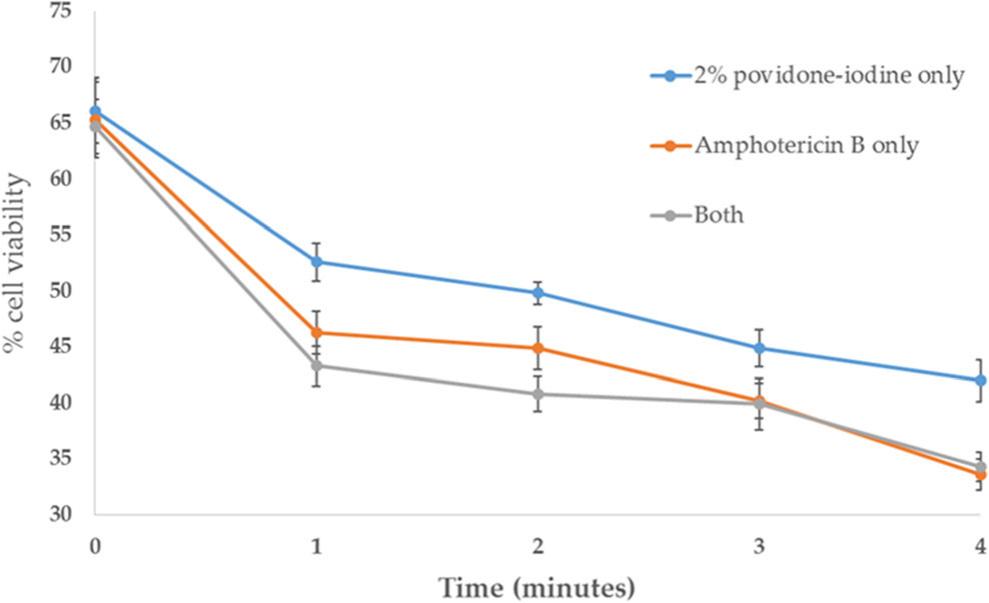
Average percentage cell viability following post-dissection disinfecting steps (*n* = 10 per disinfectant tested at each *time point*). Live/dead labelling was used to assess the viability of cells following immersion of carapace halves in 2% povidone–iodine, amphotericin B or a combination of both for 0 to 4 min. Epidermal cell viability decreased with time of exposure to the disinfectants both alone and in combination. *Error bars*: SEM.

Epidermal cell viability decreased with time of exposure to amphotericin B and/or 2% povidone–iodine (Fig. 3) over 4 min. A 13.5–21.4% decrease in viability was observed within the first minute of exposure to either of these anti-microbial agents, decreasing from 66.1% (SEM ± 2.9%), 65.3% (SEM ± 3.4%) and 64.7% (SEM ± 2.4%) to 52.6% (SEM ± 1.7%), 46.3% (SEM ± 1.9%) and 43.3% (SEM ± 1.8%) for 2% povidone–iodine, amphotericin B or a combination of both, respectively. Due to these toxic effects, these disinfectants were eliminated from our method. Instead, a strict protocol was developed using sterile ice-cold 0.9% saline, renewed for each ostracod wash with time restrictions (i.e. 2-min anaesthetisation, a second 20-s wash, before 2 min in ethanol). The ostracod was then subjected to a final wash in 0.9% ice-cold saline water (20 s) before undergoing dissection in 0.9% ice-cold saline water under a dissecting microscope. In addition, autoclaved dissection equipment and immersion of dissection tools in 2% povidone–iodine, followed by 70% ethanol, between each dissection stage was implemented.

Optimisation of Epidermal Tissue (Epidermal Explants and Carapace-Epithelial) Culture Conditions: Validation of Cell Viability and Proliferation in Culture

**Figure 4.**
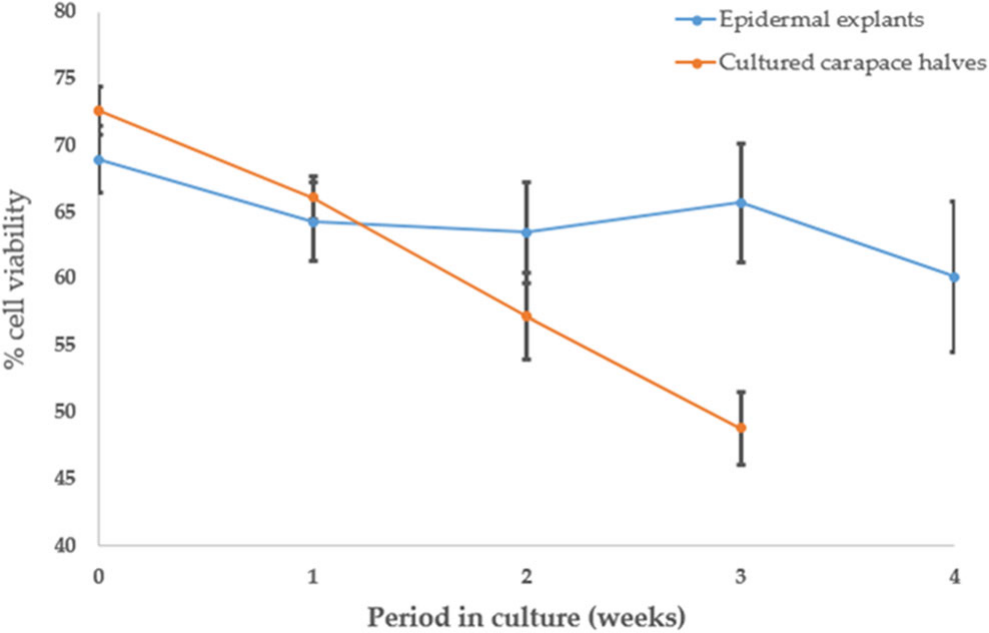
Percentage cell viability data for epidermal explant and carapace-epithelia culture (*n* = 15 per culture condition at 0 wk). Cultures in the optimised medium, which comprised a base medium of M199 with supplements, 10% FBS, 0.25 μg/ml amphotericin B, 200 μg/ml penicillin–streptomycin, 10% non-essential amino acids and 2 mM GlutaMAX, showed a high proportion of live cells at up to 4 wk in epidermal explants and up to 3 wk in carapace-epithelia. *Error bars*: SEM.

**Figure 5.**
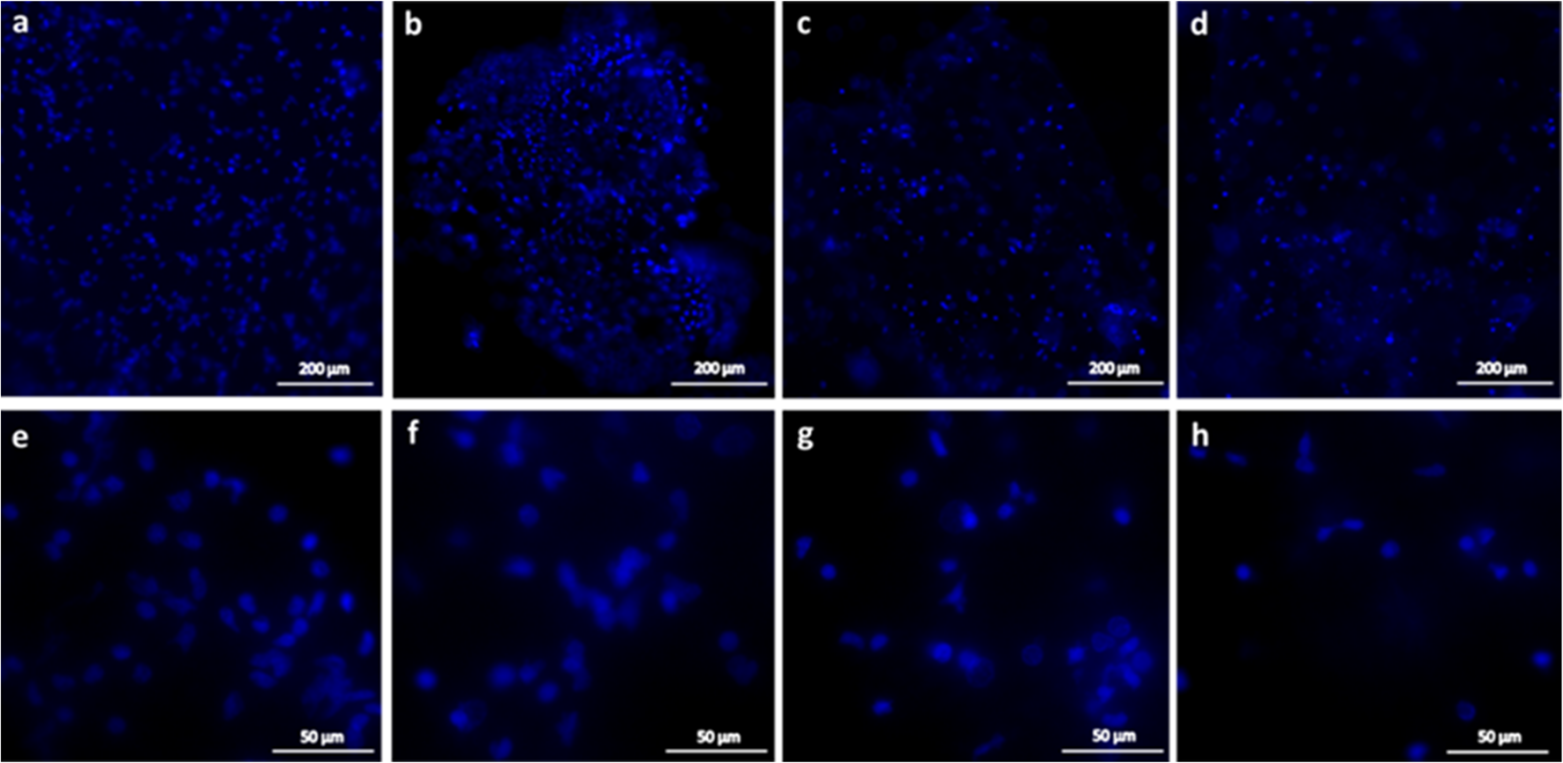
Confirmation of cells in cultured ostracod epidermal explants. Hoechst 33342 nuclear stain was used to confirm location and presence of cells after (*a*, *e*) 1, (*b*, *f*) 2, (*c*, *g*) 3 and (*d*, *h*) 4 wk in culture (*a*–*d* ×10 magnification, *e*–*h* ×40 magnification). The ostracod epidermal layer is not a flat single layer of cells and, therefore, when visualised the nuclei were in different planes and it was not possible to image all nuclei in focus.

The optimised culture conditions (based on cell viability) for *S. lerneri* carapace-epithelial tissue and epidermal explant cultures included a base medium of M199 with supplements, 10% FBS, 0.25 μg/ml amphotericin B, 200 μg/ml penicillin streptomycin, 10% non-essential amino acids and 2 mM GlutaMAX. The preferential substrate for epidermal explants was fibronectin-coated 35 mm dishes. The preferred culture environment was 25°C within a 5% CO_2_ atmosphere. These optimised conditions promoted explant adhesion and cell viability and enabled culture durations up to 4 wk with an average of 60.2% (*n* = 6, SEM ± 5.7%) cell viability for epidermal explants; and 3 wk with an average of 47.4% (*n* = 5, SEM ± 1.6%) cell viability for carapace-epithelia cultures (Fig. 4). Hoechst labelling of cell nuclei confirmed the presence and location of cells in cultured explants at each time point (Fig. 5).

**Figure 6.**
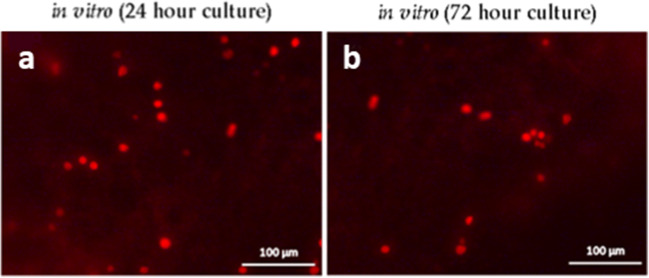
Confirmation of epidermal cell proliferation–EdU incorporation. EdU labelling in the epidermal layer of cultured carapace-epithelia following (*a*) 24 h and (*b*) 72 h of incubation. Cell proliferation was 17.0% (*n* = 5, SEM ± 1.8%) and 9.0% (*n* = 5, SEM ± 1.9%) of cells at 24 and 72 h, respectively. The ostracod carapace is a curved surface and this surface could not be flattened during sample visualisation. Therefore, when the carapace-epithelia were visualised, the nuclei were in different planes and it was not possible to image all nuclei in focus.

**Figure 7.**
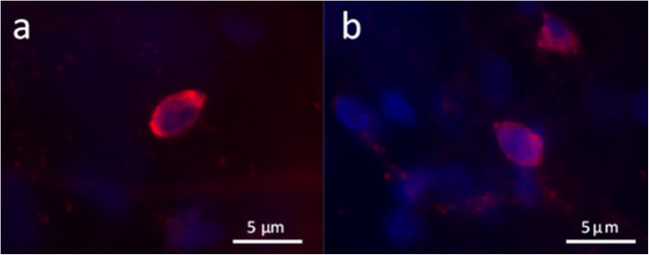
Confirmation of epidermal cell proliferation–BrdU labelling. (*a*, *b*) Fluorescent microscopy images of Rabbit anti-BrdU (ab152095) labelling (*red fluorescence*) in two of the cultured carapace-epithelia samples visualised following *in vitro* BrdU incorporation into carapace halves cultured for 48 h. Cellular proliferation was confirmed to be 14.0% (*n* = 5, SEM ± 2.8%). Cell nuclei = *blue* following Hoechst 33342 nuclear staining.

Cell proliferation assayed following EdU incorporation in the ostracod carapace-epithelia *in vitro* was 17.0% (*n* = 5, SEM ± 1.8%) and 9.0% (*n* = 5, SEM ± 1.9%) of cells at 24 h (Fig. 6*a*, *b*) and 72 h (Fig. 6*c*, *d*), respectively. Cellular proliferation was confirmed to be 14.0% (*n* = 5, SEM ± 2.8%) in the ostracod carapace-epithelia *in vitro* following BrdU incorporation into carapace halves cultured for 48 h (Fig. 7).

**Electron Microscopy of Cultured Ostracod Carapace-Epithelia**

**Figure 8.**
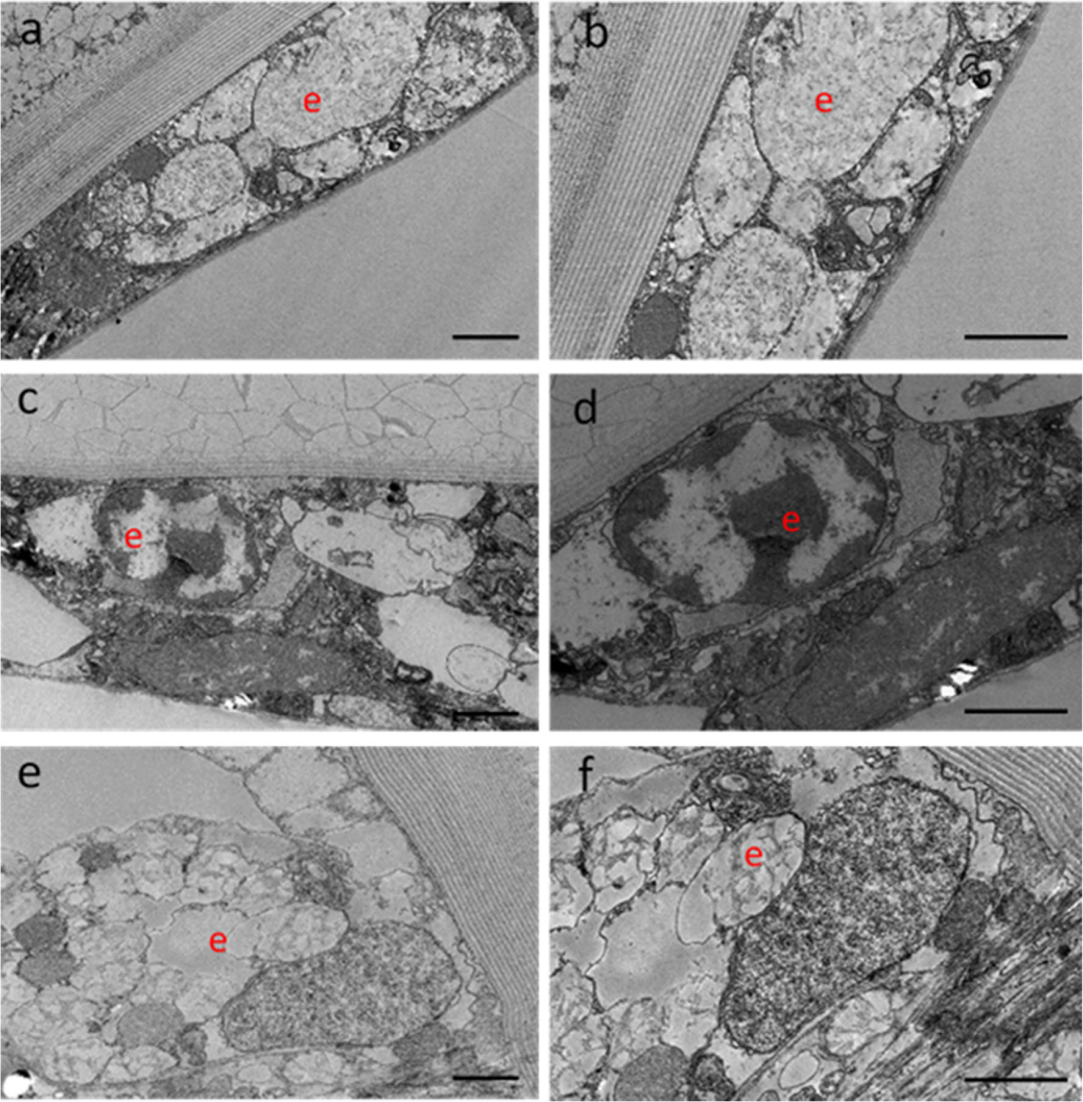
Electron microscopy of cultured epidermal tissue on adult ostracod carapaces at three culture time points (0, 1 and 2 wk, *n* = 3 at each time point). (*a*, *b*) At 0 wk in culture, the tissue is highly organised in structure compared with (*c*, *d*) 1 wk and (*e*, *f*) 2 wk. The ‘*e*’ denotes the epidermal cell layer. Images were captured at ×1200 (*left-hand column*) and ×2500 (*right-hand column*) magnification. *Scale bar*: 2 μm.

Electron microscopy of cultured carapace-epidermal tissue revealed distinct changes with culture duration (0–2 wk—Fig. 8). At 0 wk (Fig. 8*a*, *b*), the uncultured epidermal layer cells were tightly packed adjacent cells. After 1 wk in culture, cells appeared less dense (Fig. 8*c*, *d*), with gaps present. Cells were no longer in close association with the endocuticle by 2-wk culture (Fig. 8*e*, *f*).

### Epidermal Cell Culture - Optimal Dissociation Protocol for Epidermal Cell Culture

**Figure 9.**
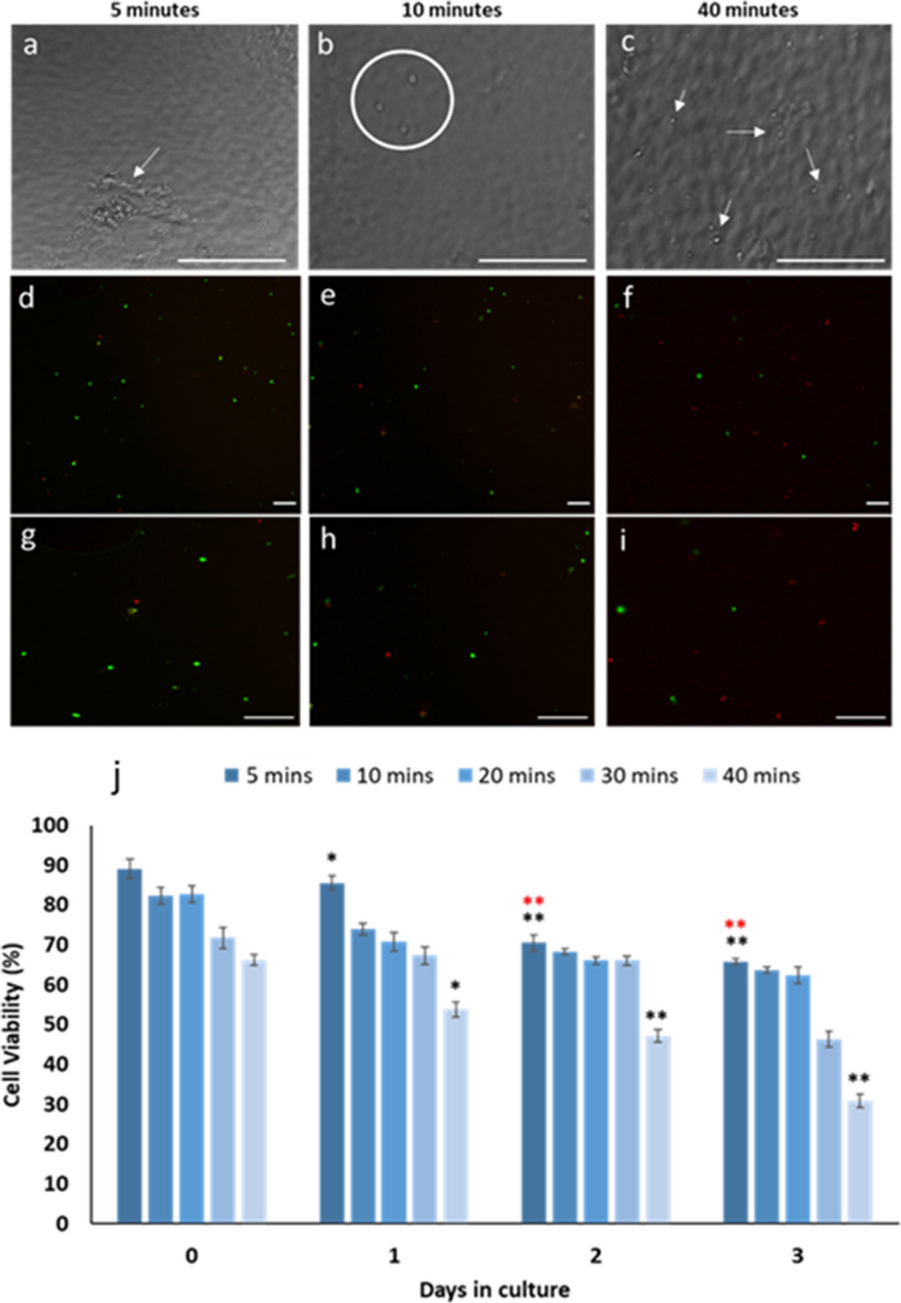
Cell dissociation using Dispase I. (*a*) Cells were in clumps following a 5-min incubation in Dispase I, with tissue remnants (*white arrows*). At (*b*) 10 min (*white circle*) and (*c*) 40 min (*white arrows*), cells were dissociated, without clustering. *Scale bars*: 20 μm Corresponding live (*green*)/dead (*red*) cell labelling is shown after (*d*, *g*) 5, (*e*, *h*) 10 and (*f*, *i*) 40-min incubations in Dispase I; (*g*)–(*i*) are magnified images of (*d*)–(*f*). *Scale bars*: 100 μm. (*j*) Percentage epidermal cell viability in culture was affected by incubation time in Dispase I; greatest cell viability in fully dissociated cells was observed after a 10-min incubation. *Error bars*: SD. *Asterisks* indicate significant differences in percentage viability between 0 d in culture (*p < 0.05, **p < 0.01) and 1 d in culture (**p < 0.01).

Cell dissociation using Dispase I in M199 (0.6–1.0 U/ml) at 25°C was determined to be the optimal concentration and temperature for isolation of cells from pooled ostracod epidermal tissue explants, resulting in complete dissociation of cells, while maintaining high cell viability (Table 1; Fig. 9*a*–*j*). After a 5-min incubation, clumps of cells and tissue remnants were observed (Fig. 9*a*), while after 10- and 40-min incubations, cell clumps were not identified, and cells appeared fully dissociated into individual cells (Fig. 9*b*, *c*) and were viable (Fig. 9*e*, *f*, *h*, *i*). However, epidermal cell viability in culture was affected by duration time of the Dispase I incubation (Fig. 9), especially after a 40-min incubation with a decline in cell viability at 1 d (*p* < 0.05), 2 d (*p* < 0.01) and 3 d (*p* < 0.01) in culture compared with the 0-d culture, from 65.8% (*n* = 3, SD ± 0.8%) at day 0 to 30.9% (*n* = 3, SD ± 1.7%) on culture day 3. In addition, a significant decrease in cell viability was observed at culture days 1 (*p* < 0.05), 2 (*p* < 0.01) and 3 (*p* < 0.01) in cells subjected to a 5-min Dispase I incubation. The latter may be a result of retention of clumped cells and tissue remnants at this 5-min incubation time. Since complete dissociation of cells (Fig. 9*b*, *e*, *h*) was achieved at 10 min, while maintaining the cell viability over all cultures at all time points, this was deemed the optimal Dispase I incubation time.

### Epidermal Cell Culture - Optimal Dissociation Protocol for Epidermal Cell Culture

**Figure 10.**
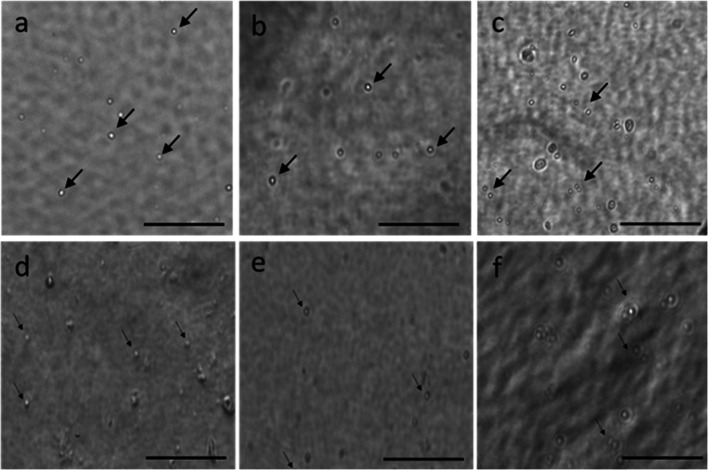
Phase-contrast images of dissociated cells adhering to substrates (*a*) laminin, (*b*) fibronectin, (*c*) collagen type IV, (*d*) chitosan, (*e*) Matrigel and (*f*) collagen type I. A larger number of cells (arrows) was observed adhered to collagen type I, while cell spreading was most apparent on collagen type IV. Scale bars: 20 μm.

Dissociated cells adhered to all substrates tested by culture day 7 (Fig. 10). Cells on laminin (Fig. 10*a*), chitosan (Fig. 10*d*), collagen type IV (Fig. 10*c*) and collagen type I (Fig. 10*f*) were more easily identified and appeared greatest in number, compared with those on fibronectin (Fig. 10*b*) and Matrigel (Fig. 10*e*) coated plates. In addition, cell spreading was observed on substrates collagen I and IV, with the greatest number of spread cells present on collagen IV (Fig. 10*c*). Based on cell adhesion, viability and spreading, collagen I and IV were selected as optimal substrates for culture of dissociated cells and further tested to determine if cell proliferation occurred in culture.

### Epidermal Cell Proliferation

**Figure 11.**
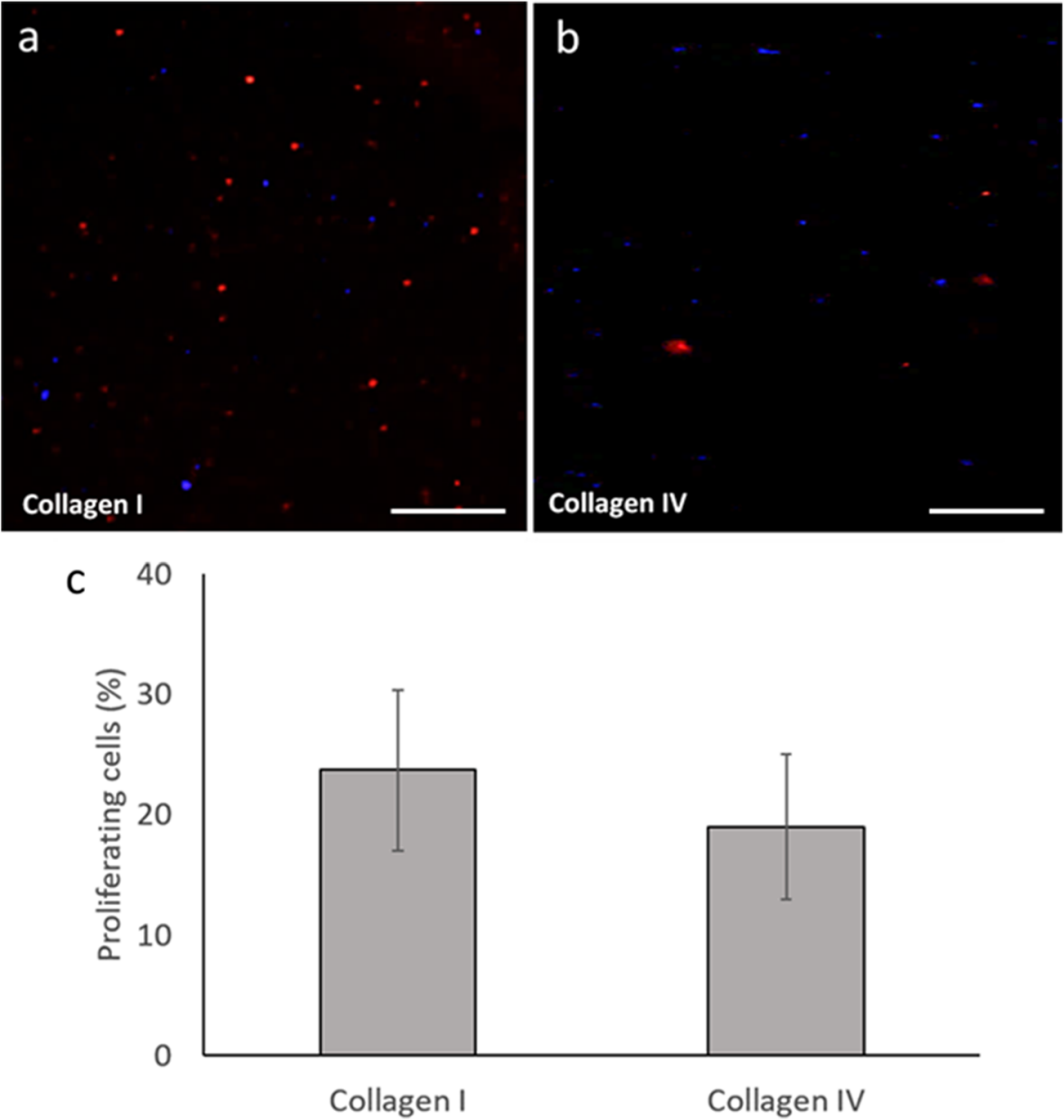
Ostracod epidermal cell proliferation in dissociated cells at 7 d of culture. (*a*, *b*) Proliferating cells (BrdU labelled; *red fluorescence*) expressed as a percentage of total cells (Hoechst-labelled nuclei; *blue fluorescence*) on substrates, collagen types I and IV, were quantified. *Scale bars*: 50 μm. (*c*) Cell proliferation was not found to be significantly different on these two substrates following 7 d in culture (*p* = 0.211). *Error bars*: SD.

Percentage epidermal cell proliferation assayed following BrdU incorporation after 7 d of culture on collagen type I (Fig. 11*a*) and type IV (Fig. 11*b*) coated plates was 23.7% (*n* = 3, SD ± 6.0%) and 19.0% (*n* = 3, SD ± 6.7%), respectively (Fig. 11*c*).

## Discussion

Crustacean cell culture has acquired recognition as a compelling model to aid in the production of diagnostic reagents and probes for use in the shrimp, crayfish and lobster industries (Toullec 1999). Hundreds of cell lines from insects and mammals have been developed; however, despite numerous attempts, current marine crustacean cell lines reported on the Cellosaurus resource database are limited to two SV-40 transformed cell lines of *Penaeus stylirostris* (Pacific blue shrimp) (OKTr-23 (RRID:CVCL_9U41) and OKTr-1 (RRID:CVCL_9U40); Tapay *et al*. 1995). To date, no ostracod-derived cell lines have been reported.

The aim of this work was to determine the best methods for sample preparation and the culture of ostracod *S. lerneri* epidermal tissue (either as carapace-epidermal tissue or explants) and epidermal cells (i.e. cells dissociated from explants). In order to develop a cell culture medium optimised for these cultures, reliable methods of assessing cell viability and proliferation were required. The live/dead labelling assay used provided a simple, fast and quantitative method of evaluating cell viability in order to determine the effectiveness of the medium and/or supplements combination to support cell longevity in culture.

Primary cells introduced into culture are susceptible to contamination, but antibiotics or antimicrobials may limit, but not prevent, the growth of contaminants (Fogh *et al*. 1971). Yeast and fungal contaminations are especially problematic; therefore, aseptic primary culture techniques are essential. Optimisation of removal of viable epidermal tissue for culture was achieved, despite a number of potentially detrimental processes, including anaesthetisation, decontamination, epidermal dissection and culture protocols, while preventing contamination of cultures. Decontamination of ostracod carapace halves with amphotericin B and/or 2% povidone–iodine had considerable toxic effects on epidermal cell viability even before culture. Therefore, stringent protocols to eliminate contaminant exposure were developed to ensure epidermal cell viability for ostracod cultures of epidermal explants, carapace epidermal tissue cultures and cell cultures.

Thereafter, a variety of media and an extensive range of supplements, including nutrients, growth factors, chemical elements, inorganic compounds and amino acids, were trialled to develop an optimal culture medium. Primary cell cultures from ostracods have yet to be developed; therefore, knowledge of potential media and supplements were sourced from the literature reporting on other attempts of crustacean cell culture. These were predominantly of shrimp primary cell cultures, attained from a number of organ sources, including epidermal cultures (Chen *et al*. 1986; Luedeman and Lightner 1992; Toullec and Dauphin-Villemant 1994; Hsu *et al*. 1995; Frerichs 1996; Toullec *et al*. 1996; Kasornchandra *et al*. 1999; Owens and Smith 1999; Toullec 1999; Walton and Smith 1999; Mulford *et al*. 2001; Shimizu *et al*. 2001; Lang *et al*. 2002a; Lang *et al*. 2002b; Maeda *et al*. 2003). Of the base media tested, M199, which contains a wide variety of vitamins and amino acids, prolonged epidermal tissue survival in culture. This was consistent with M199, along with L-15, being used in the successful culture of crustacean primary cells from various tissues (Brody and Chang 1989; Luedeman and Lightner 1992; Nadala *et al*. 1993; Ghosh *et al*. 1995; Hsu *et al*. 1995; Frerichs 1996; Toullec *et al*. 1996; Chen and Wang 1999; Itami *et al*. 1999; Owens and Smith 1999; Shimizu *et al*. 2001; Lang *et al*. 2002a, 2002b; Maeda *et al*. 2003). Inclusion of low concentrations of amphotericin B and penicillin–streptomycin within the media successfully prevented fungal and bacterial contaminations.

Thereafter, an extensive range of supplements, including nutrients, growth factors, chemical elements, inorganic compounds and amino acids, were trialled to develop an optimal culture medium. Of the supplements tested, FBS, non-essential amino acids and GlutaMAX were found to enhance primary epidermal cell survival in both tissue and cell culture. The inclusion of FBS was based on it being a highly complex component used frequently to supplement media with minerals, lipids and hormones (Ma *et al*. 2017), as well as providing growth and adhesion factors to promote cell attachment and proliferation (Fang *et al.* 2017). It is unlikely to provide all of the characteristic hormones and growth factors required by crustacean cells (Mulford *et al*. 2001), although for *S. lerneri* epidermal cell culture it proved to be adequate for cell survival. Non-essential amino acids were added, as the balance of amino acids in crustacean cultures can impact on cell survival and growth rate (Shimizu *et al*. 2001).

The inclusion of GlutaMAX was essential as a source of carbon and energy (Butler and Christie 1994), as well as being able to improve cell survival (Pasieka and Morgan 1959). GlutaMAX was used over l-glutamine as it does not degrade spontaneously in culture or generate toxic ammonia which can limit cell growth (Schneider *et al*. 1996) and is known to be toxic to life in aquaculture.

In contrast to previous studies of crustacean culture systems in which supplements, such as NE salts (Shimizu *et al*. 2001), HEPES buffer (Luedeman and Lightner 1992; Hsu *et al*. 1995; Toullec 1999; Lang *et al*. 2002a), NaCl (Chen et al. 1986; Hsu *et al*. 1995; Frerichs 1996; Kasornchandra *et al*. 1999; Walton and Smith 1999; Shimizu *et al*. 2001) and bFGF (Hsu *et al*. 1995), increased cell viability and longevity, these made no contribution to *S. lerneri* epidermal cultures.

Ostracod epidermal cells proliferated in cultures of carapace-epidermal tissue, explants and cells, demonstrating cell survival following tissue dissection and/or isolation. The decline in proliferation over culture duration may be indicative of initial stress or ‘culture shock’ activation of cell proliferation when placed in culture (Sherr and DePinho 2000; Lang *et al*. 2004) followed by a period of acclimatisation. The percentage of proliferating cells was similar in all tissue and cell cultures, supporting the use of this optimised medium for epidermal cell survival.

In addition, the proliferative state of cells may be impacted by the ostracod moult stage. Typically, ostracods develop through a series of juvenile instars via a moulting process (ecdysis) which ends when the mature adult instar is reached (Smith *et al*. 2015). The moult cycle of crustaceans is described in stages: postmoult, intermoult, premoult and ecdysis. In an ostracod lifecycle, the younger instars moult more rapidly than the more mature animals and there is substantial individual variation in instar duration (Turpen and Angell 1971). Some crustaceans (e.g. decapods) spend much of their cycle in the intermoult stage (Passano 1960), where epithelial cells in contact with the cuticle display cellular inactivity (Green and Neff 1972; Chassard-Bouchaud and Hubert 1973; Hubert and Chassard-Bouchaud 1978). It is only during sub-stages of premoult where epithelial mitotic activity becomes apparent (Skinner 1962, 1966; Stevenson 1972; Hubert and Chassard-Bouchaud 1978). It is conceivable that ostracod epithelial cells are only mitotically active for defined and varying time periods during their lifecycle. Further work is required to confirm the proliferative state of epithelial cells at different moult stages of *S. lerneri*.

## Conclusions

The present study is the first to describe the culture of viable ostracod epidermal tissue and cells. Having established a method of maintaining the epidermal layer and cells *in vitro*, with cell proliferation, there is potential to explore possible ways of mimicking carapace synthesis in three-dimensional culture. The knowledge of this complex construction could then be used to replicate its manufacture at an industrial level to assemble materials where transparency, durability and strength are prerequisites for their functional use. Progress towards establishing marine crustacean cell lines also has positive ramifications for the field of *in vitro* toxicity testing. Therefore, future research will be focused on extending the survival time of primary ostracod cells and the development of an immortal cell line for sustained and indefinite expansion *in vitro*.
